# Attitudes towards cardiopulmonary resuscitation situations and associations with potential influencing factors—A survey among in-hospital healthcare professionals

**DOI:** 10.1371/journal.pone.0271686

**Published:** 2022-07-15

**Authors:** Jennie Silverplats, Anneli Strömsöe, Björn Äng, Marie-Louise Södersved Källestedt

**Affiliations:** 1 School of Health and Welfare, Dalarna University, Falun, Sweden; 2 Department of Anaesthesiology and Intensive Care, Region Dalarna, Mora Hospital, Mora, Sweden; 3 Center for Clinical Research Dalarna, Uppsala University, Falun, Sweden; 4 Department of Prehospital Care, Region Dalarna, Falun, Sweden; 5 Division of Physiotherapy, Department of Neurobiology, Care Sciences and Society, Karolinska Institutet, Huddinge, Sweden; 6 Centre for Clinical Research Västmanland, Uppsala University, Västerås, Sweden; Beth Israel Deaconess Medical Center / Harvard Medical School, UNITED STATES

## Abstract

**Introduction:**

Attitudes towards cardiopulmonary resuscitation (CPR) among in-hospital healthcare professionals (HCPs) are poorly understood. The aim of this study was to evaluate attitudes towards CPR situations among in-hospital HCPs and assess associations with potential influencing factors.

**Materials and methods:**

A questionnaire was distributed to 3,085 HCPs in 2009 and 2,970 HCPs in 2015–2016. The associations of influencing factors were analyzed using binary logistic regression.

**Results:**

In the event of a possible cardiac arrest situation, 61% of the HCPs would feel confident in their CPR knowledge, 86% would know what to do, and 60% would be able to take command if necessary. In the latest real-life CPR situation, 30% had been worried about making mistakes or causing complications, 57% had been stressed, and 27% had been anxious. A short time since the latest real-life CPR performance and a high number of previous real-life CPR performances were associated with lower odds of worrying about making mistakes/causing complications, lower odds of feeling stressed or anxious, and higher odds of feeling calm. Regardless of previous real-life CPR experience, there were differences in attitudes between groups of professions, where physicians showed increased odds of worrying about making mistakes/causing complications and nurses showed increased odds of stress. Working on a non-monitored ward meant increased odds of stress and worrying about making mistakes/causing complications. Twelve months or more having passed since the latest CPR training course was associated with increased odds of anxiety.

**Conclusions:**

Despite HCPs’ generally positive attitudes towards performing CPR in the event of a possible cardiac arrest situation, feelings of stress and anxiety were common in real-life CPR situations. Regular CPR training among all HCPs is a key factor to maintain competence and reduce anxiety. The possible effects of attitudes on performing CPR need to be studied further.

## Introduction

A cardiac arrest requires immediate treatment, with any delay decreasing the chance of patient survival [1]. The attitudes of healthcare professionals (HCPs) towards cardiopulmonary resuscitation (CPR) situations could affect their behavior in such situations. Attitude is seen as an essential component of competence [2] and is, among laymen, the strongest predictor of the intention to perform CPR [3, 4]. Attitude consists of positive or negative feelings, beliefs, and behavioral information [5]. Among HCPs, cardiac arrest situations have previously been associated with feelings of stress [6, 7], hesitation [8, 9], anxiety [8, 10], and fear of harming the patient [8]. Previous real-life CPR experience has been associated with a positive attitude towards CPR [11]. Previous research has often been based on small samples and focused on specific professions. Factors associated with attitudes are poorly understood. Performing CPR is a team effort, and the attitudes among all professions possibly involved in a cardiac arrest situation are important. There is a lack of large studies among all in-hospital professions regarding attitudes towards CPR situations and including assessment of potentially associated factors. The aim of this study was to evaluate attitudes among in-hospital HCPs towards CPR situations and assess associations with possible influencing factors.

## Materials and methods

### Design, setting and data collection

**Fig 1:**
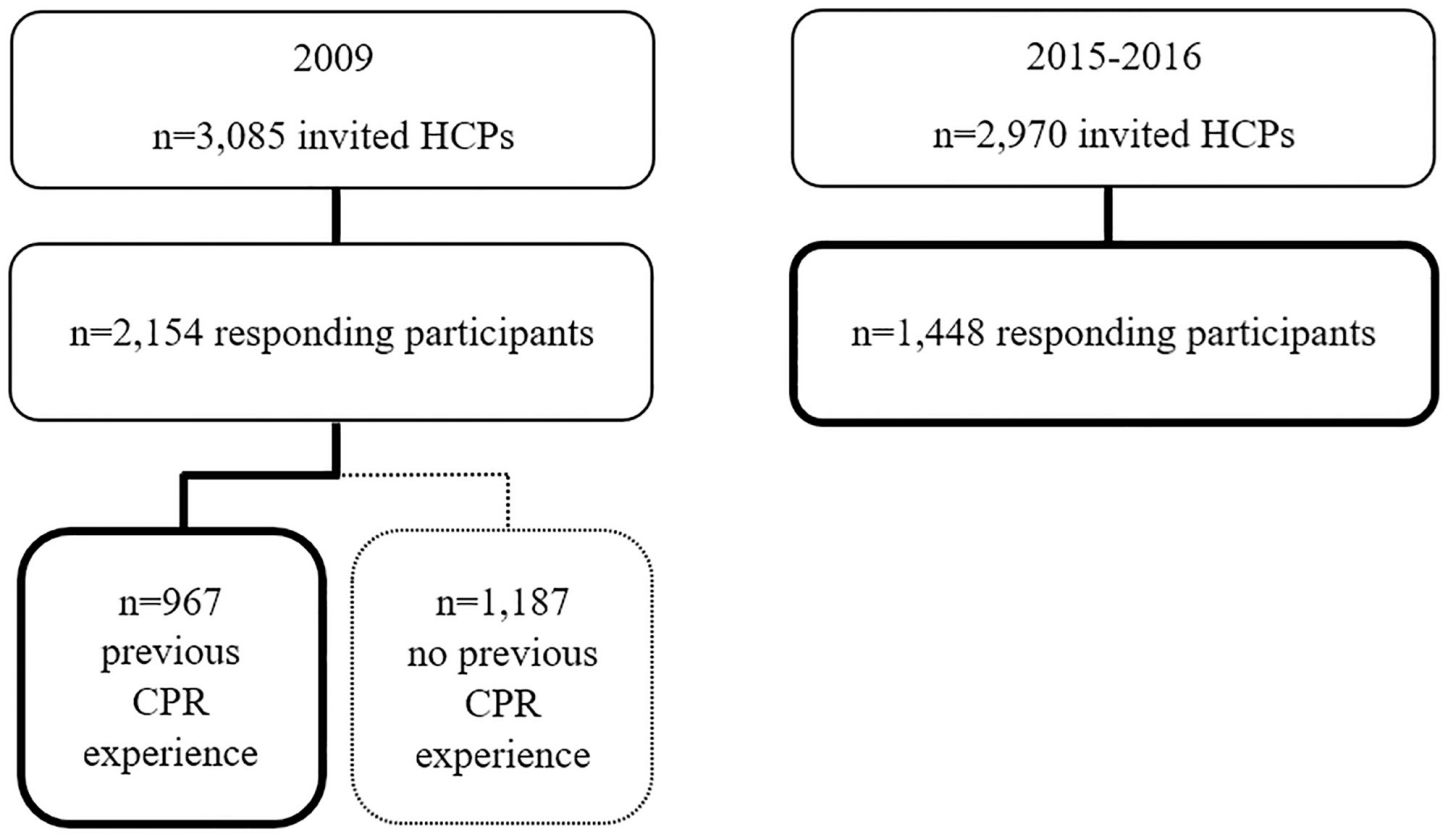
Flowchart of participants.

Two cross-sectional surveys were performed in two regions in Sweden during 2009 [12] and 2015–2016 [13] at a total of five hospitals. The focus of the survey in 2009 was the impact of organized CPR training on attitudes towards CPR and CPR situations. Data were collected two years after implementation of organized CPR training at two secondary care hospitals. During 2015–2016, data concerning theoretical knowledge, self-assessed ability and attitudes towards CPR were collected among HCPs at three secondary care hospitals with organized CPR training. The training was structured in a similar way at all hospitals, in accordance with guidelines from the Swedish Resuscitation Council. The data were collected one year before a 5-year update of international guidelines in both regions. The hospitals varied in size from 45 to 600 in-patient beds on both monitored and non-monitored wards. All active-duty HCPs involved in patient contact were invited to participate. In 2009, 3,085 HCPs were invited, and in 2015–2016, 2,970 HCPs were invited (Fig 1), for a total of 6,055 invited HCPs. The HCPs were physicians, nurses (including midwifes and radiology nurses), nursing assistants (including caretakers and dental nurses) and other university-educated staff (physiotherapists, occupational therapists, social welfare officers, psychologists, biomedical analysts, dentists, audiologists, speech therapists, dieticians and counsellors). Participation was anonymous in one region and confidential in the other. HCPs were informed of the study at staff meetings or by e-mail. The questionnaires were distributed either at staff meetings or to mailboxes on the wards in one region and directly to participating individuals in the other region. Reminders were sent by e-mail. In-hospital CPR training was provided by the employer. It encompassed CPR and use of automated external defibrillators (AEDs) on non-monitored wards and advanced CPR on monitored wards. The training requirements given by the Swedish Resuscitation Council target all HCPs, regardless of profession or workplace. All HCPs should be able to treat a sudden cardiac arrest with CPR and use a semi-automatic defibrillator. CPR training should be attended every six months or at least once a year [14].

### The questionnaire

The validity and reliability of the questionnaire were evaluated through interviews with HCPs, by consulting experts in CPR and pedagogy, and through a test-retest procedure among HCPs [12]. Twenty-one questions, presented in S1 File, were evaluated in this study. The questions not included in this study have been analyzed elsewhere [12, 13]. All questions were multiple choice. In 2009, the participants responded to four questions regarding previous real-life CPR experience: experience of performing CPR on a child/adult in a cardiac arrest situation (yes/no), number of previous real-life CPR performances (one to more than 30) and time since the latest real-life CPR performance (one month to 24 months or more). Participants who reported having previous real-life CPR experience responded to eight additional questions concerning attitude towards the latest cardiac arrest situation. In 2015–2016, the participants responded to six questions concerning attitudes towards being required to perform CPR in the event of a cardiac arrest situation. In both surveys, all participants responded to three questions concerning occupation, number of years in profession, and time since latest CPR training course. Each participant’s workplace was noted during the collection of the questionnaires. Electronic scanning combined with visual checking minimized human error in data entry.

### Statistics

The results from the attitude questions are presented descriptively. Differences regarding attitudes between groups of professions were analyzed using Pearson’s chi-squared test (significance level *P*<.05). The association of possible influencing factors were analyzed using binary logistic regression. Each of the eight questions concerning attitudes during the latest cardiac arrest situation (yes/no) served as a dependent variable, where odds ratios for the response ‘yes’ were estimated. Independent variables were number of previous real-life CPR performances (1–3, 4–10, or more than 10), time since latest real-life CPR performance (24 months or more, 4–23 months, or 0–3 months), workplace (monitored versus non-monitored ward), profession (physician, nurse, assistant nurse, or other university-educated staff), time since latest CPR training course (0–6 months, 7–11 months, or 1 year ago or more/never), and number of years in profession (from 0 to 46 years). Each independent variable was regressed with the dependent variables in a crude model, one at a time, and only independent variables which were found to be associated (*P*<0.1) were included in multiple models. Independent variables considered not to be associated (*P*>0.1) were excluded from the multiple models. In the final multiple model, independent variables that were considered to be associated with the dependent variable at a level of *P*<.05 were considered statistically significant. Data were analyzed using IBM Statistical Package for the Social Sciences, version 27. A consensus-based checklist for reporting of survey studies (CROSS) was used for reporting [15].

### Ethics approval and consent to participate

This study was given an additional amendment by the ethical review authority in Uppsala (Dnr 2006/201/2) and was registered at ClinicalTrials.gov (ID nr NCT04321213). HCPs consented to participation by responding to the questionnaires.

## Results

In 2009, 70% (n = 2,154) of the HCPs participated in the survey, of whom 45% (n = 967) stated that they had previous real-life CPR experience. In 2015–2016, 49% (n = 1,448) of the HCPs responded. The total response rate was 59% (n = 3,602). The rate of missing items was low, ranging from 0.1% to 7.2%. Mean work experience was 19.0 years (2009) and 18.6 years (2015–2016). There were low attendance rates at CPR training: 33% (2009) and 45% (2015–2016). Thirty-five percent of the respondents in 2009 worked on monitored wards versus 20% of the respondents in 2015–2016. In 2009, 42% (n = 401/967) had performed CPR one to three times and 47% (n = 457/967) had most recently performed CPR more than two years ago (Table 1).

**Table 1:** Occupation, CPR training, previous CPR experience and workplace.

	2009					2015–2016				
	Physicians	Nurses	Assistant nurses	Other university-educated staff	Total	Physicians	Nurses	Assistant nurses	Other university-educated staff	Total
	*n = 208*	*n = 545*	*n = 197*	*n = 15*	***n = 965*** ^1^	*n = 265*	*n = 605*	*n = 427*	*n = 150*	***n = 1*,*447*** ^2^
**Years in profession** *Mean (SD)*	19.0 (10.9)	17.5 (11.1)	23.0 (10.8)	19.9 (11.5)	19.0 (11.2)	14.4 (11.4)	18.8 (12.3)	21.7 (14.1)	16.9 (11.8)	18.6 (12.9)
**CPR**^3^ **training ≤ 11 months ago**	26 (13)	195 (36)	95 (48)	3 (20)	319 (33)	96 (36)	328 (54)	195 (46)	34 (23)	653 (45)
**Monitored ward**	26 (12)	241 (44)	72 (36)	0 (0)	339 (35)	13 (5)	178 (29)	100 (23)	-	291 (20)
**Non-monitored ward**	182 (88)	304 (56)	125 (64)	15 (100)	626 (65)	252 (95)	427 (71)	327 (77)	150 (100)	1,156 (80)
**No. of CPR performances**										
**1–3**	46 (23)	230 (43)	113 (64)	12 (79)	401 (42)	n.a.	n.a.	n.a.	n.a.	
**4–10**	73 (36)	153 (29)	44 (22)	2 (13)	272 (28)	n.a.	n.a.	n.a.	n.a.	
**>10**	84 (42)	144 (28)	26 (14)	1 (6)	255 (26)	n.a.	n.a.	n.a.	n.a.	
**Time since latest CPR performance**										
**0–3 months**	52 (24)	141 (26)	24 (13)	0 (0)	217 (22)	n.a.	n.a.	n.a.	n.a.	
**4–23 months**	32 (15)	155 (29)	69 (37)	2 (14)	258 (27)	n.a.	n.a.	n.a.	n.a.	
**≥24 months**	122 (59)	230 (44)	92 (50)	13 (87)	457 (47)	n.a.	n.a.	n.a.	n.a.	

The table shows the samples from the surveys in 2009 and 2015–2016. The respondents in 2009, n = 967, had real-life CPR experience. Numbers are presented as within-group frequencies, n (%).

^1^ missing n = 2.

^2^ missing n = 1.

^3^ cardiopulmonary resuscitation.

### Attitudes towards cardiopulmonary resuscitation situations

In the event of a possible cardiac arrest situation, the majority of HCPs would feel confident in their CPR knowledge (61%), know what to do (86%), and be able to take command if necessary (60%). Still, 42% of HCPs would feel nervous and 26% anxious. There were significant differences between groups of professions. Other university-educated staff showed attitudes considered to be negative, e.g., fewer reported confidence in their CPR knowledge and more reported anxiety in the event of a cardiac arrest situation, compared with other professions (Table 2).

**Table 2:** Attitudes towards being required to perform CPR in the event of a cardiac arrest situation.

	Physicians	Nurses	Assistant nurses	Other university-educated staff	Total	Missing	*P*
	*n = 265*	*n = 605*	*n = 427*	*n = 150*	*n = 1*,447*	*n*	
*I would be unsure of my reaction*	60 (23)	129 (21)	83 (19)	95 (63)	367 (25)	22	<.001
*I would feel nervous*	147 (56)	236 (39)	134 (31)	93 (62)	610 (42)	27	<.001
*I would feel confident in my CPR knowledge*	159 (60)	409 (68)	268 (63)	39 (26)	875 (61)	23	<.001
*I would know what to do in the event of a cardiac arrest*	244 (92)	548 (91)	360 (84)	86 (57)	1238 (86)	24	<.001
*I would feel anxious*	81 (31)	137(23)	82 (19)	73 (49)	373 (26)	31	<.001
*I would take command of the situation if necessary*	224 (85)	418 (69)	173 (41)	56 (37)	871 (60)	20	<.001

The table shows those who responded ‘yes’ to the respective questions. Numbers are presented as within-group frequencies, n (%).

* missing n = 1.

Only fifteen among the other university-educated staff had previous real-life CPR experience; they were found to be outliers in the multiple models. The following results are therefore only applicable to physicians, nurses, and assistant nurses. Table 3 shows attitudes regarding performing CPR in connection to the latest real-life cardiac arrest situation.

**Table 3:** Attitudes towards performing CPR in connection to the latest real-life cardiac arrest situation.

	Physicians	Nurses	Assistant nurses	Total	Missing	*P*
	*n = 208*	*n = 545*	*n = 197*	*n = 950* *	*n*	
**During the latest real-life cardiac arrest situation, did you feel…**						
*Worried about contracting an illness*	16 (7)	24 (4)	14 (7)	54 (5)	43	.09
*Worried about making mistakes or causing complications*	72 (35)	157 (29)	52 (26)	281 (30)	46	.12
*Discomfort at initiating CPR*	30 (14)	56 (10)	14 (7)	100 (11)	72	.09
**After the latest real-life cardiac arrest situation, did you feel…**						
*Stressed*	94 (45)	336 (62)	113 (57)	543 (57)	45	.01
*Anxious*	55 (26)	144 (26)	59 (30)	258 (27)	49	.35
*Calm*	98 (47)	288 (53)	109 (55)	495 (52)	53	.31
*Like a failure*	29 (14)	58 (11)	16 (8)	103 (11)	48	.07
*Pleased*	75 (36)	273 (50)	115 (53)	463 (49)	50	<.001

The table shows those who responded ‘yes’ to the respective questions. Numbers are presented as within-group frequencies, n (%).

* missing n = 2, other university-educated staff excluded (n = 15).

During the latest cardiac arrest situation, 30% of HCPs were worried about making mistakes or causing complications, but few were worried about contracting an illness (5%) or felt discomfort at initiating CPR (11%). After the latest cardiac arrest situation, 57% of HCPs reported feeling stressed, 27% reported feeling anxious, 52% reported feeling calm, 49% reported feeling pleased, and 11% reported feeling like a failure. Significant differences between groups of professions were found regarding perceived stress and feeling pleased with the performance in the situation (Table 4).

**Table 4:** Factors associated with attitudes in connection to the latest real-life cardiac arrest situation.

Factor	Worried about making mistakes or causing complications	Feeling anxious	Feeling stressed	Feeling calm	Feeling pleased
Time since latest real-life CPR* **performance**					
*0–3 months*	0.41 (0.24, 0.70) *P* = .001	0.26 (0.15, 0.47) *P*<.001	0.24 (0.15, 0.38) *P*<.001	5.11 (2.94, 8.89) *P*<.001	N.A.
*4–23 months*	0.74 (0.49, 1.13) *P* = .162	0.83 (0.57, 1.23) *P* = .352	0.53 (0.34, 0.82) *P* = .005	1.45 (0.98, 2.15) *P* = .067	N.A.
*≥ 24 months (ref)*	1	1	1	1	N.A.
**Number of real-life CPR performances**					
*>10*	0.10 (0.05, 0.21) *P*<.001	0.24 (0.17, 0.34) *P*<.001	0.32 (0.21, 0.50) *P*<.001	2.72 (1.63, 4.53) *P*<.001	2.10 (1.42, 3.16) *P* = .001
*4–10*	0.81 (0.55, 1.19) *P* = .289	0.24 (0.17, 0.34) *P*<.001	1.74 (1.08, 2.80) *P* = .023	0.95 (0.63, 1.42) *P* = .796	1.45 (1.01, 2.11) *P* = .047
*1–3 (ref)*	1	1	1	1	1
**Workplace (ref. monitored ward)**	1.55 (1.04, 2.30) *P* = .032	N.A.	1.76 (1.19, 2.61) *P* = .005	N.A.	N.A.
**Occupation**					
*Physician*	2.14 (1.34, 3.42) *P* = .001	N.A.	0.31 (0.19, 0.50) *P*<.001	0.94 (0.59, 1.47) *P* = .774	0.48 (0.33, 0.70) *P*<.001
*Assistant nurse*	0.84 (0.54, 1.30) *P* = .430	N.A.	0.44 (0.28, 0.69) *P*<.001	2.06 (1.30, 3.26) *P* = .002	2.10 (1.37, 3.23) *P* = .001
*Nurse (ref)*	1	N.A.	1	1	1
**Years in profession (per year)**	0.97 (0.95, 0.99) *P* = .001	N.A.	N.A.	N.A.	N.A.
**Time since latest CPR course**					
*1 year ago or more/never*	N.A.	1.90 (1.15, 3.15) *P* = .012	N.A.	N.A.	N.A.
*7–11 months*	N.A.	1.16 (0.61, 2.20) *P* = .645	N.A.	N.A.	N.A.
*0–6 months (ref)*	N.A.	1	N.A.	N.A.	N.A.

The table shows odds ratios with confidence intervals.

*Cardiopulmonary resuscitation.

### Associated factors

Table 4 shows the results of the final adjusted multiple logistic models regarding factors associated with attitudes in connection to the latest real-life CPR situation. All crude and multiple models are presented in S1 Table. A time of 0–3 months since the latest real-life CPR performance and having performed real-life CPR more than 10 times previously were associated with lower odds of being worried about making mistakes or causing complications, lower odds of feeling stressed or anxious, and higher odds of feeling calm. Regardless of the amount of previous real-life CPR experience, there were associations between occupation and attitudes. Physicians showed increased odds of worrying about making mistakes or causing complications compared with nurses. The odds of feeling stressed were lower among physicians and assistant nurses compared with nurses. The odds of feeling pleased were lower among physicians and higher among assistant nurses compared with nurses. Assistant nurses showed higher odds of feeling calm compared with nurses. Regardless of previous real-life CPR experience or occupation, workplace (monitored or non-monitored ward) was a significant factor for being worried about making mistakes or causing complications and for feeling stressed, where HCPs working on non-monitored wards showed higher odds. Regardless of the above factors, number of years in the profession was associated with being worried about making mistakes or causing complications, with each year meaning lower odds. The time since the latest CPR training course was associated only with feeling anxious, where CPR training 1 year ago or more/never was associated with increased odds regardless of previous real-life CPR experience (Table 4). Worrying about contracting an illness during CPR, feelings of discomfort at initiating CPR, and feelings of failure after CPR performance were not possible to analyze in multiple models due to few observations and should be interpreted with caution (S1 Table).

## Discussion

The main findings in this study were that a majority of HCPs felt confident in their CPR knowledge, would know what to do, and would take command if necessary in a cardiac arrest situation. A third of the HCPs reported being worried about making mistakes or causing complications. Stress and anxiety were common in connection to the latest real-life cardiac arrest situation. A short time since the latest real-life CPR performance and a high number of previous real-life CPR performances were associated with lower odds of worrying about making mistakes/causing complications, lower odds of feeling stressed or anxious, and higher odds of feeling calm. Regardless of previous real-life CPR experience, there were differences in attitudes between groups of professions and between HCPs on monitored and non-monitored wards. Twelve months or more having passed since the latest CPR training course was associated with increased odds of anxiety.

In contrast of previous research, this large survey evaluated attitudes towards CPR situations among a wide range of in-hospital professions. Worry, stress, and anxiety are closely related, but differ as regards the individual’s physical response. Worry is a cognitive problem-solving process, not generally associated with physiological arousal [16]. Stress derives from a perceived challenge or threat that generates a physiological response. A challenge is perceived as manageable and generates a positive physiological response, whereas a threat generates a negative physiological response and affective state, most commonly anxiety [17]. A cardiac arrest situation is potentially highly stressful, and stress could either enhance or impair performance in high-acuity situations [17]. A perceived threatful situation or stress overload means that an individual’s resources are insufficient relative to the demands. Stress overload affects mental or cognitive functions, such as attention, memory, and decision-making, and can have a negative impact on teamwork and management of a sudden cardiac arrest [17, 18]. In this study, anxiety was reported among 26% of HCPs when required to perform CPR, and among 27% after the latest real-life CPR performance. Also, 57% of the HCPs reported feeling stressed. Self-reported stress is a good marker of perceived mental stress and is strongly associated with impaired performance in acute emergency settings [18]. Perceived stress has been proven to delay initiation of CPR in simulated situations [19] and only a third of first responders managed to treat a cardiac arrest correctly in a simulated situation [20]. Failure to build an initial team structure was common [20]. The Swedish CPR guidelines are based on European and international guidelines derived from the chain of survival concept. The chain regarding early, high-quality CPR requires that all HCPs who are involved in patient care can perform CPR [21]. It is well-known that delayed initiation of CPR is associated with worse patient outcome [1, 22]. Regular CPR training among all HCPs is therefore of great importance. In the present study, only 33% and 45% of all HCPs in 2009 and 2015–2016, respectively, had attended CPR training within the preceding year.

The results in the present study showed differences between groups of professions regarding attitudes, regardless of previous real-life CPR experience. This could be due to predefined roles and responsibilities in the cardiac arrest situation. Nurses had higher odds of stress compared with physicians and assistant nurses. Nurses are most often bedside first responders and are responsible for the initial treatment together with assistant nurses, before the arrival of the emergency team. Nurses have described sudden cardiac arrest situations as initially chaotic with both physical and mental reactions including feelings of panic and tunnel vision [6]. Simulated situations show that quick and directive leadership among first responders during the initial phase enhances team performance [23]. Since 2010, the European Resuscitation Council recommends leadership training in advanced CPR training courses [24]. However, leadership training among nurses trained in CPR AED has been associated with improved initial treatment [25]. Given the importance of the treatment during the first minutes of a cardiac arrest, it seems beneficial to teach leadership in basic in-hospital CPR training courses. Other university-educated staff showed a low compliance to training requirements and reported attitudes considered as negative in a possible cardiac arrest situation. These professions are rarely involved in cardiac arrest situations, but their competence is an important contribution to the chain of survival. There is a need to increase compliance to training requirements among these professions. A majority of HCPs on monitored wards attend advanced CPR training and working in areas at high risk of in-hospital cardiac arrest has been shown to be associated with increased confidence in performing CPR and being part of a resuscitation team [26]. In this study, a difference in attitude was seen between HCPs on monitored wards and those on non-monitored wards, regardless of previous real-life CPR experience and occupation. This suggests that advanced CPR training might be associated with lower odds of stress and lower odds of worry about making mistakes or causing complications compared with CPR AED training.

CPR training should be performed with a frequency of at least once a year [27]. Stress coping styles, having a feeling of control, and receiving social support are factors that affect an individual’s stress response and performance [17]. Also, understanding and awareness of stress response [17], the use of cognitive aids and checklists [27, 28], and post-arrest debriefing [6, 27, 29] are considered important for stress management. In-hospital performance-focused debriefing is recommended for teams managing patients in cardiac arrest in the 2015 European Resuscitation Council guidelines and among all rescuers in the 2021 guidelines [27], but the compliance in Swedish hospitals is unknown. An individual’s response to stressful events can be influenced by changing the perception of demands and resources of emergency situations [30], preferably through instructions or discussions during training (briefing) or debriefing [27]. In this way, the situation can be perceived as a challenge rather than as a threat, which could significantly impact on clinical performance. The results in this study imply a need for increased compliance to CPR training requirements among all HCPs. Regular attendance in refresher courses maintains competence and provides the resources needed to manage a cardiac arrest situation.

There are limitations in this study. There were differences in the focus and time intervals of the surveys. Both surveys were conducted among HCPs who attended similar training one year before a 5-year change in international guidelines. It is not possible to measure any differences in attitudes regarding time and focus due to a cross-sectional design with subjective responses from different individuals. Therefore, the samples were presented separately. Cross-sectional studies have limitations: they can reveal possible associations, but causality cannot be evaluated as cause and effect are studied simultaneously. It is also possible that there are confounders not controlled for in this study. Non-responders could lead to selection bias. The proportion of non-responders in this study may introduce a serious bias. Due to anonymity, it was not possible to evaluate the non-responders in 2015–2016. The total number of distributed questionnaires and the number of questionnaires distributed to each group of professions were based on manager information regarding the number of employees. Some of the HCPs rotated between workplaces. The higher response rate in 2009 was probably due to the confidential approach, which made it possible to send individual reminders. Competence and quality of CPR skills have previously been reported to be overestimated by HCPs. In this attitude study, there might be a risk that some HCPs chose the answer that was expected of them or accepted by others in their role as an HCP. The large samples in this study are representative of the study population. However, HCPs from teaching hospitals were not included in the sample, and their attitudes are presently unknown. There is a need for future research regarding attitudes towards CPR and CPR situations among in-hospital HCPs. Possible barriers or facilitators and their effects on real-life CPR performance need to be explored.

## Conclusions

Despite HCPs’ generally positive attitudes towards performing CPR in the event of a possible cardiac arrest situation, feelings of stress and anxiety were common in real-life CPR situations. Regular CPR training among all HCPs is a key factor to maintain competence and reduce anxiety. The possible effects of attitudes on performing CPR need to be studied further.

## Supporting information

S1 File Included questions. (DOCX)

S1 Table Associations of influencing factors—Crude and multiple models. (DOCX)
