# Supplementary material for: Attitudes towards cardiopulmonary resuscitation situations and associations with potential influencing factors—A survey among in-hospital healthcare professionals
Source: PLoS One. 2022 Jul 15;17(7):e0271686. doi: 10.1371/journal.pone.0271686 (PMC9286263; doi:10.1371/journal.pone.0271686)
Supplement: S1 File — (DOCX) [file pone.0271686.s001.docx]

**S1. Included questions.**

**Attitudes when being required to perform CPR**

Options: Yes, No, I don’t know

You are on your way to work, and you are aware of that there are patients treated for heart disease on your ward. There is an increased risk of a cardiac arrest, and you might be required to perform CPR. How do you feel about that?

| 1. I would be unsure of my reaction |
| --- |
| 2. I would feel nervous |
| 3. I would feel confident in my CPR knowledge |
| 4. I would know what to do in the event of a cardiac arrest |
| 5. I would feel anxious |
| 6. I would take command of the situation if necessary |

**Demographic information**

| 7. Please provide information of your professional occupation: | |
| --- | --- |
|  | Nursing assistant, Care taker |
|  | Nurse, Midwife |
|  | Biomedical analyst |
|  | Physiotherapist |
|  | Occupational therapist |
|  | Audiologist |
|  | Physician |
|  | Other profession, please specify……………………………………… |

| 8. For how long have you been working in healthcare? | \|  \|  \| years \| \| --- \| --- \| --- \| |
| --- | --- | --- | --- | --- |

| 9. How long ago did you participate in CPR training? | | | | | | |
| --- | --- | --- | --- | --- | --- | --- |
| Never | Last month | 2-3 months | 4-6 months | 7-11 months | 12-23 months | ≥24 months |
|  |  |  |  |  |  |  |

**Previous real-life CPR experience**

| 10. Have you ever performed CPR on a child in a real-life cardiac arrest situation? | |
| --- | --- |
| Yes | No |
|  |  |

| 11. Have you ever performed CPR on an adult in a real-life cardiac arrest situation? | | | | | | |  |  |
| --- | --- | --- | --- | --- | --- | --- | --- | --- |
| Yes | | | No | | | |  |  |
|  | | |  | | | |  |  |
|  | | | | | | | | |
| 12. How many times have you performed CPR in a real-life cardiac arrest situation? | | | | | | | | |
| 1 time | 2-3 times | 4-5 times | | 6-10 times | 11-20 times | 21-30 times | | > 30 times |
|  |  |  | |  |  |  | |  |

| 13. How long ago did you perform CPR in a real-life cardiac arrest situation? | | | | | | |
| --- | --- | --- | --- | --- | --- | --- |
| Never | Last month | 2-3 months | 4-6 months | 7-11 months | 12-23 months | ≥24 months |
|  |  |  |  |  |  |  |

**Attitudes in connection to the latest cardiac arrest situation**

Options: Yes very, Yes a bit, No, I don’t recall

During the latest cardiac arrest situation, did you feel:

| 14. Worried to contract an illness? |
| --- |
| 15. Worried about making mistakes or causing complications? |
| 16. Discomfort in initiating CPR? |

After the latest cardiac arrest situation, did you feel

| 17. Stressed? |
| --- |
| 18. Anxious? |
| 19. Calm? |
| 20. Like a failure? |
| 21. Pleased? |
