# Supplementary material for: Attitudes towards cardiopulmonary resuscitation situations and associations with potential influencing factors—A survey among in-hospital healthcare professionals
Source: PLoS One. 2022 Jul 15;17(7):e0271686. doi: 10.1371/journal.pone.0271686 (PMC9286263; doi:10.1371/journal.pone.0271686)
Supplement: S1 Table — (DOCX) [file pone.0271686.s002.docx]

|  | **Crude model** | | | **Multiple model 1** | | | **Multiple model 2** | | |
| --- | --- | --- | --- | --- | --- | --- | --- | --- | --- |
|  | **OR** | **CI** | ***p*** | **aOR** | **CI** | ***p*** | **aOR** | **CI** | ***p*** |
| **Worried about contracting an illness** |  |  |  |  |  |  |  |  |  |
| Time since last real-life CPR performance |  |  |  |  |  |  |  |  |  |
| 0-3 months | 1.02 | 0.51, 2.04 | 0.951 | - | - | - |  |  |  |
| 4-23 months | 1.16 | 0.61, 2.19 | 0.654 | - | - | - |  |  |  |
| ≥24 months (Ref) | - | - | - | - | - | - |  |  |  |
| Number of real-life CPR performances |  |  |  |  |  |  |  |  |  |
| >10 times | 0.79 | 0.39, 1.57 | 0.496 | - | - | - |  |  |  |
| 4-10 times | 0.95 | 0.49, 1.82 | 0.882 | - | - | - |  |  |  |
| 1-3 times (Ref) | - | - | - | - | - | - |  |  |  |
| Time since last CPR course |  |  |  |  |  |  |  |  |  |
| ≥ 12 months | 1.36 | 0.56, 3.31 | 0.502 | - | - | - |  |  |  |
| 7-11 months | 3.07 | 1.18, 8.02 | 0.022 | - | - | - |  |  |  |
| 0-6 months (Ref) |  |  |  | - | - | - |  |  |  |
| Workplace (Ref monitored ward) | 1.07 | 0.61, 1.90 | 0.811 | - | - | - |  |  |  |
| Professional occupation |  |  |  |  |  |  |  |  |  |
| Physicians | 1.91 | 0.99, 3.69 | 0.052 | - | - | - |  |  |  |
| Assistant nurses | 1.74 | 0.88, 3.43 | 0.113 | - | - | - |  |  |  |
| Other univ. Educ. staff | - | - | - | - | - | - |  |  |  |
| Nurses (Ref) | - | - | - | - | - | - |  |  |  |
| Years in profession | 0.99 | 0.97, 1.02 | 0.580 | - | - | - |  |  |  |
|  |  |  |  |  |  |  |  |  |  |
|  |  |  |  |  |  |  |  |  |  |
|  |  |  |  |  |  |  |  |  |  |
| **Worried about making mistakes or to cause complications** |  |  |  |  |  |  |  |  |  |
| Time since last real-life CPR performance |  |  |  |  |  |  |  |  |  |
| 0-3 months | 0.37 | 0.25, 0.54 | < 0.001 | 0.54 | 0.33, 0.86 | 0.010 | 0.41 | 0.24, 0.70 | 0.001 |
| 4-23 months | 0.65 | 0.46, 0.91 | 0.012 | 0.78 | 0.53, 1.15 | 0.202 | 0.74 | 0.49, 1.13 | 0.162 |
| ≥24 months (Ref) | - | - | - | - | - | - | - | - | - |
| Number of real-life CPR performances |  |  |  |  |  |  |  |  |  |
| >10 times | 0.26 | 0.18, 0.39 | < 0.001 | 0.31 | 0.19, 0.51 | < 0.001 | 0.10 | 0.05, 0.21 | < 0.001 |
| 4-10 times | 0.74 | 0.53, 1.04 | 0.083 | 0.82 | 0.57, 1.19 | 0.301 | 0.81 | 0.55, 1.19 | 0.289 |
| 1-3 times (Ref) | - | - | - | - | - | - | - | - | - |
| Time since last CPR course |  |  |  |  |  |  |  |  |  |
| ≥ 12 months | 1.16 | 0.79, 1.71 | 0.461 | - | - | - | - | - | - |
| 7-11 months | 0.745 | 0.45, 1.24 | 0.254 | - | - | - | - | - | - |
| 0-6 months (Ref) | - | - | - | - | - | - | - | - | - |
| Workplace (Ref monitored ward) | 2.07 | 1.52, 2.82 | < 0.001 | 1.36 | 0.95, 1.95 | 0.098 | 1.55 | 1.04, 2.30 | 0.032 |
| Professional occupation |  |  |  |  |  |  |  |  |  |
| Physicians | 1.40 | 0.98, 1.98 | 0.063 | 1.53 | 1.01, 2.32 | 0.046 | 2.14 | 1.34, 3.42 | 0.001 |
| Assistant nurses | 0.93 | 0.64, 1.36 | 0.716 | 0.83 | 0.54, 1.25 | 0.365 | 0.84 | 0.54, 1.30 | 0.430 |
| Other univ. Educ. staff | 3.41 | 1.10, 10.60 | 0.034 | 2.13 | 0.67, 6.82 | 0.203 | - | - | - |
| Nurses (Ref) | - | - | - | - | - | - | - | - | - |
| Years in profession | 0.98 | 0.96, 0.99 | 0.001 | 0.98 | 0.96, 0.99 | 0.009 | 0.97 | 0.95, 0.99 | 0.001 |
|  |  |  |  |  |  |  |  |  |  |
|  |  |  |  |  |  |  |  |  |  |
|  | **Crude model** | | | **Multiple model 1** | | | **Multiple model 2** | | |
|  | **OR** | **CI** | ***p*** | **aOR** | **CI** | ***p*** | **aOR** | **CI** | ***p*** |
| **Discomfort in initiating CPR** |  |  |  |  |  |  |  |  |  |
| Time since last real-life CPR performance |  |  |  |  |  |  |  |  |  |
| 0-3 months | 0.39 | 0.22, 0.70 | 0.002 | - | - | - | - | - | - |
| 4-23 months | 0.53 | 0.32, 0.88 | 0.015 | - | - | - | - | - | - |
| ≥24 months (Ref) | - | - | - | - | - | - | - | - | - |
| Number of real-life CPR performances |  |  |  |  |  |  |  |  |  |
| >10 times | 0.19 | 0.09, 0.37 | < 0.001 | - | - | - | - | - | - |
| 4-10 times | 0.46 | 0.28, 0.76 | 0.002 | - | - | - | - | - | - |
| 1-3 times (Ref) | - | - | - | - | - | - | - | - | - |
| Time since last CPR course |  |  |  |  |  |  |  |  |  |
| ≥ 12 months | 1.17 | 0.67, 2.05 | 0.585 | - | - | - | - | - | - |
| 7-11 months | 0.70 | 0.32, 1.53 | 0.370 | - | - | - | - | - | - |
| 0-6 months (Ref) | - | - | - | - | - | - | - | - | - |
| Workplace (Ref monitored ward) | 1.97 | 1.23, 3.16 | 0.005 | - | - | - | - | - | - |
| Professional occupation |  |  |  |  |  |  |  |  |  |
| Physicians | 1.45 | 0.90, 2.35 | 0.127 | - | - | - | - | - | - |
| Assistant nurses | 0.72 | 0.39, 1.34 | 0.301 | - | - | - | - | - | - |
| Other univ. Educ. staff | 1.54 | 0.39, 7.21 | 0.584 | - | - | - | - | - | - |
| Nurses (Ref) | - | - | - | - | - | - | - | - | - |
| Years in profession | 0.99 | 0.97, 1.01 | 0.180 | - | - | - | - | - | - |
|  |  |  |  |  |  |  |  |  |  |
|  |  |  |  |  |  |  |  |  |  |
|  |  |  |  |  |  |  |  |  |  |
|  |  |  |  |  |  |  |  |  |  |
| **Feeling stressed** |  |  |  |  |  |  |  |  |  |
| Time since last real-life CPR performance |  |  |  |  |  |  |  |  |  |
| 0-3 months | 0.26 | 0.18, 0.37 | < 0.001 | 0.40 | 0.26, 0.62 | < 0.001 | 0.24 | 0.15, 0.38 | < 0.001 |
| 4-23 months | 0.68 | 0.48, 0.98 | 0.036 | 0.82 | 0.55, 1.22 | 0.322 | 0.53 | 0.34, 0.82 | 0.005 |
| ≥24 months (Ref) | - | - | - | - | - | - | - | - | - |
| Number of real-life CPR performances |  |  |  |  |  |  |  |  |  |
| >10 times | 0.25 | 0.18, 0.36 | < 0.001 | 0.35 | 0.23, 0.54 | < 0.001 | 0.32 | 0.21, 0.50 | < 0.001 |
| 4-10 times | 1.07 | 0.74, 1.56 | 0.715 | 1.34 | 0.89, 2.02 | 0.165 | 1.74 | 1.08, 2.80 | 0.023 |
| 1-3 times (Ref) | - | - | - | - | - | - | - | - | - |
| Time since last CPR course |  |  |  |  |  |  |  |  |  |
| ≥ 12 months | 1.40 | 0.96, 2.05 | 0.082 | 1.33 | 0.86, 2.04 | 0.202 | - | - | - |
| 7-11 months | 1.40 | 0.87, 2.26 | 0.167 | 1.47 | 0.86, 2.15 | 0.166 | - | - | - |
| 0-6 months (Ref) | - | - | - | - | - | - | - | - | - |
| Workplace (Ref monitored ward) | 1.72 | 1.29, 2.29 | < 0.001 | 1.40 | 0.97, 2.01 | 0.070 | 1.76 | 1.19, 2.61 | 0.005 |
| Professional occupation |  |  |  |  |  |  |  |  |  |
| Physicians | 0.59 | 0.41, 0.84 | 0.004 | 0.53 | 0.34, 0.82 | 0.004 | 0.31 | 0.19, 0.50 | < 0.001 |
| Assistant nurses | 0.91 | 0.63, 1.32 | 0.623 | 0.66 | 0.47, 0.99 | 0.049 | 0.44 | 0.28, 0.69 | < 0.001 |
| Other univ. Educ. staff | 2.38 | 0.52, 10.99 | 0.266 | 1.21 | 0.25, 5.80 | 0.810 | - | - | - |
| Nurses (Ref) | - | - | - | - | - | - | - | - | - |
| Years in profession | 0.99 | 0.98, 1.01 | 0.463 | - | - | - | - | - | - |
|  |  |  |  |  |  |  |  |  |  |
|  |  |  |  |  |  |  |  |  |  |
|  |  |  |  |  |  |  |  |  |  |
|  | **Crude model** | | | **Multiple model 1** | | | **Multiple model 2** | | |
|  | **OR** | **CI** | ***p*** | **aOR** | **CI** | ***p*** | **aOR** | **CI** | ***p*** |
| **Feeling anxious** |  |  |  |  |  |  |  |  |  |
| Time since last real-life CPR performance |  |  |  |  |  |  |  |  |  |
| 0-3 months | 0.25 | 0.17, 0.39 | < 0.001 | 0.53 | 0.32, 0.87 | 0.011 | 0.26 | 0.15, 0.47 | < 0.001 |
| 4-23 months | 0.61 | 0.43, 0.86 | 0.005 | 0.89 | 0.60, 1.30 | 0.530 | 0.83 | 0.57, 1.23 | 0.352 |
| ≥24 months (Ref) | - | - | - | - | - | - | - | - | - |
| Number of real-life CPR performances |  |  |  |  |  |  |  |  |  |
| > 10 times | 0.15 | 0.09, 0.24 | < 0.001 | 0.16 | 0.09, 0.33 | < 0.001 | 0.24 | 0.17, 0.34 | < 0.001 |
| 4-10 times | 0.49 | 0.35, 0.69 | < 0.001 | 0.51 | 0.35, 0.74 | < 0.001 | 0.24 | 0.17, 0.34 | < 0.001 |
| 1-3 times (Ref) | - | - | - | - | - | - | - | - | - |
| Time since last CPR course |  |  |  |  |  |  |  |  |  |
| ≥ 12 months | 1.55 | 1.01, 2.36 | 0.043 | 1.55 | 0.97, 2.47 | 0.070 | 1.90 | 1.15, 3.15 | 0.012 |
| 7-11 months | 1.17 | 0.69, 1.97 | 0.567 | 1.41 | 0.79, 2.51 | 0.249 | 1.16 | 0.61, 2.20 | 0.645 |
| 0-6 months (Ref) | - | - | - | - | - | - | - | - | - |
| Workplace (Ref monitored ward) | 1.98 | 1.44, 2.73 | < 0.001 | 1.13 | 0.77, 1.65 | 0.523 | - | - | - |
| Professional occupation |  |  |  |  |  |  |  |  |  |
| Physicians | 1.13 | 0.78, 1.65 | 0.516 | 1.35 | 0.86, 2.12 | 0.190 | - | - | - |
| Assistant nurses | 1.31 | 0.90, 1.90 | 0.161 | 0.99 | 0.66, 1.49 | 0.972 | - | - | - |
| Other univ. Educ. staff | 4.74 | 1.40, 15.98 | 0.012 | 2.63 | 0.74, 9.37 | 0.135 | - | - | - |
| Nurses (Ref) | - | - | - | - | - | - | - | - | - |
| Years in profession | 0.99 | 0.98, 1.00 | 0.170 | - | - | - | - | - | - |
|  |  |  |  |  |  |  |  |  |  |
|  |  |  |  |  |  |  |  |  |  |
|  |  |  |  |  |  |  |  |  |  |
|  |  |  |  |  |  |  |  |  |  |
| **Feeling calm** |  |  |  |  |  |  |  |  |  |
| Time since last real-life CPR performance |  |  |  |  |  |  |  |  |  |
| 0-3 months | 4.23 | 2.77, 6.46 | < 0.001 | 3.23 | 1.99, 5.23 | < 0.001 | 5.11 | 2.94, 8.89 | < 0.001 |
| 4-23 months | 1.75 | 1.23, 2.48 | 0.002 | 1.48 | 1.02, 2.16 | 0.042 | 1.45 | 0.98, 2.15 | 0.067 |
| ≥24 months (Ref) | - | - | - | - | - | - | - | - | - |
| Number of real-life CPR performances |  |  |  |  |  |  |  |  |  |
| >10 times | 2.84 | 1.92, 4.22 | < 0.001 | 2.01 | 1.27, 3.18 | 0.003 | 2.72 | 1.63, 4.53 | < 0.001 |
| 4-10 times | 1.07 | 0.75, 1.52 | 0.715 | 0.93 | 0.64, 1.36 | 0.707 | 0.95 | 0.63, 1.42 | 0.796 |
| 1-3 times (Ref) | - | - | - | - | - | - | - | - | - |
| Time since last CPR course |  |  |  |  |  |  |  |  |  |
| ≥ 12 months | 0.69 | 0.45, 1.05 | 0.086 | 0.82 | 0.52, 1.30 | 0.397 | - | - | - |
| 7-11 months | 0.97 | 0.58, 1.64 | 0.915 | 0.88 | 0.50, 1.53 | 0.639 | - | - | - |
| 0-6 months (Ref) | - | - | - | - | - | - | - | - | - |
| Workplace (Ref monitored ward) | 0.56 | 0.41, 0.77 | < 0.001 | 0.88 | 0.60, 1.23 | 0.494 | - | - | - |
| Professional occupation |  |  |  |  |  |  |  |  |  |
| Physicians | 0.89 | 0.61, 1.30 | 0.560 | 0.98 | 0.63, 1.52 | 0.918 | 0.94 | 0.59, 1.47 | 0.774 |
| Assistant nurses | 1.28 | 0.86, 190 | 0.229 | 1.62 | 1.06, 2.48 | 0.025 | 2.06 | 1.30, 3.26 | 0.002 |
| Other univ. Educ. staff | 0.16 | 0.005, 0.67 | 0.011 | 0.31 | 0.08, 1.18 | 0.086 | - | - | - |
| Nurses (Ref) | - | - | - | - | - | - | - | - | - |
| Years in profession | 1.00 | 0.99, 1.01 | 0.996 | - | - | - | - | - | - |
|  |  |  |  |  |  |  |  |  |  |
|  |  |  |  |  |  |  |  |  |  |
|  |  |  |  |  |  |  |  |  |  |
|  | **Crude model** | | | **Multiple model 1** | | | **Multiple model 2** | | |
|  | **OR** | **CI** | ***p*** | **aOR** | **CI** | ***p*** | **aOR** | **CI** | ***p*** |
| **Feeling pleased** |  |  |  |  |  |  |  |  |  |
| Time since last real-life CPR performance |  |  |  |  |  |  |  |  |  |
| 0-3 months | 1.01 | 0.71, 1.46 | 0.941 | - | - | - | - | - | - |
| 4-23 months | 0.98 | 0.69, 1.38 | 0.890 | - | - | - | - | - | - |
| ≥24 months (Ref) | - | - | - | - | - | - | - | - | - |
| Number of real-life CPR performances |  |  |  |  |  |  |  |  |  |
| >10 times | 1.44 | 1.01, 2.06 | 0.045 | 1.85 | 1.26, 2.71 | 0.002 | 2.10 | 1.42, 3.16 | < 0.001 |
| 4-10 times | 1.21 | 0.85, 1.71 | 0.283 | 1.40 | 0.97, 2.02 | 0.71 | 1.45 | 1.01, 2.11 | 0.047 |
| 1-3 times (Ref) | - | - | - | - | - | - | - | - | - |
| Time since last CPR course |  |  |  |  |  |  |  |  |  |
| ≥ 12 months | 0.95 | 0.65, 1.41 | 0.811 | - | - | - |  |  |  |
| 7-11 months | 1.15 | 0.70, 1.89 | 0.572 | - | - | - |  |  |  |
| 0-6 months (Ref) | - | - | - | - | - | - |  |  |  |
| Workplace (Ref monitored ward) | 0.97 | 0.72, 1.31 | 0.825 | - | - | - |  |  |  |
| Professional occupation |  |  |  |  |  |  |  |  |  |
| Physicians | 0.57 | 0.39, 0.82 | 0.002 | 0.49 | 0.34, 0.72 | < 0.001 | 0.48 | 0.33, 0.70 | < 0.001 |
| Assistant nurses | 1.68 | 1.12, 2.50 | 0.011 | 1.78 | 1.18, 2.69 | 0.006 | 2.10 | 1.37, 3.23 | 0.001 |
| Other univ. Educ. staff | 0.73 | 0.74, 2.21 | 0.579 | 0.86 | 0.28, 2.64 | 0.798 | - | - | - |
| Nurses (Ref) | - | - | - | - | - | - | - | - | - |
| Years in profession | 0.99 | 0.99, 1.01 | 0.812 | - | - | - | - | - | - |
|  |  |  |  |  |  |  |  |  |  |
| **Feelings of failure** |  |  |  |  |  |  |  |  |  |
| Time since last real-life CPR performance |  |  |  |  |  |  |  |  |  |
| 0-3 months | 0.45 | 0.24, 0.83 | 0.011 | - | - | - | - | - | - |
| 4-23 months | 1.21 | 0.77, 1.90 | 0.422 | - | - | - | - | - | - |
| ≥24 months (Ref) | - | - | - | - | - | - | - | - | - |
| Number of real-life CPR performances |  |  |  |  |  |  |  |  |  |
| >10 times | 0.34 | 0.19, 0.60 | < 0.001 | - | - | - | - | - | - |
| 4-10 times | 0.64 | 0.40, 1.04 | 0.069 | - | - | - | - | - | - |
| 1-3 times (Ref) | - | - | - | - | - | - | - | - | - |
| Time since last CPR course |  |  |  |  |  |  |  |  |  |
| ≥ 12 months | 0.88 | 0.52, 1.51 | 0.643 | - | - | - | - | - | - |
| 7-11 months | 0.86 | 0.44, 1.71 | 0.675 | - | - | - | - | - | - |
| 0-6 months (Ref) | - | - | - | - | - | - | - | - | - |
| Workplace (Ref monitored ward) | 1.61 | 1.03, 2.52 | 0.037 | - | - | - | - | - | - |
| Professional occupation |  |  |  |  |  |  |  |  |  |
| Physicians | 1.50 | 0.92, 2.44 | 0.102 | - | - | - | - | - | - |
| Assistant nurses | 0.73 | 0.41, 1.31 | 0.295 | - | - | - | - | - | - |
| Other univ. Educ. staff | 3.57 | 1.04, 12. 23 | 0.043 | - | - | - | - | - | - |
| Nurses (Ref) |  |  |  | - | - | - | - | - | - |
| Years in profession | 0.99 | 0.97, 1.01 | 0.414 |  |  |  | - | - | - |
|  |  |  |  |  |  |  |  |  |  |
